# Principles and Methods for Evidence-Based Quantification of the Effect of Seat Belt Non-Use in Crash-Related Litigation

**DOI:** 10.3390/ijerph18189455

**Published:** 2021-09-08

**Authors:** Michael D. Freeman

**Affiliations:** CAPHRI School for Public Health and Primary Care, Faculty of Health, Medicine, and Life Sciences, Maastricht University, 6211 LM Maastricht, The Netherlands; m.freeman@maastrichtuniversity.nl

**Keywords:** road traffic crash, seat belt, risk, NASS-CDS, INFERENCE causation

## Abstract

Traffic crashes are a common cause of injury and death, and often result from the negligent actions of an inattentive, speeding, or impaired driver. In such cases, a civil legal action may be brought by an injured claimant for compensation for injuries resulting from a crash. Crash-related litigation is defended on various theories, one of which is to raise the issue of contributory negligence when the claimant was not using an available seat belt at the time of the crash, based on the assertion that the claimed injuries would have been avoided or minimized to some degree if the claimant had been restrained. At present, there are no published standards or systematic approach for assessing and quantifying the contribution of seat belt non-use to the cause of a claimant’s specific injury. A reliable medicolegal analysis that addresses whether contributory negligence can be proven in a specific case requires a multidisciplinary approach: First, the nature and severity of the crash must be reconstructed as it affected the vehicle kinetics (engineering) and in turn affected the kinematics of the occupant (biomechanics), next, the injuries must be described and scaled for severity (medicine/pathology), and finally, the risk of the known injuries given the actual circumstances of the crash and occupant (i.e., unbelted) are compared to the risk of the same injuries, and the same crash circumstances, but in the hypothetical scenario in which the claimant is belted. In the present discussion, methods for analyzing the presence and quantifying the degree of contributory negligence for seat belt non-use, suitable for presentation in a medicolegal setting, are described and illustrated with an example from the author’s personal case inventory. A detailed reconstruction of the crash is described, along with the associated occupant kinematics, and the resulting observed injuries. The injuries are then categorized by their anatomical location, type, and severity using Abbreviated Injury Scale designations. Quantification of the injury risk for the actual (unbelted) vs. hypothetical (belted) scenario is based on case-specific analysis of data accessed from a US national crash injury database The difference in risk for the two exposure scenarios can be quantified in terms of either relative risk (a risk ratio) or attributable risk (a risk proportion), with the goal to determine whether the analysis meets the threshold of a relative risk of >2.0, or an attributable risk of 50%, in order to meet the “more probable than not” standard typically required by courts. As a final step in a reliable analysis that exceeds the legal threshold for relevant evidence, the absolute increase in risk is used to quantify the degree to which the claimant’s seat belt non-use contributed to the likelihood of their injuries.

## 1. Introduction

Road traffic crashes are the most common cause of serious injury and death in the US and Europe [1]. In 2020, the World Health Organization estimated the worldwide annual toll of traffic crashes of 1.35 million deaths, and between 20 and 50 million injuries [2]. Traffic crashes are primarily caused by the negligent actions of a driver, including inattention/distraction, excessive speed, and impairment due to the effects of intoxicants [3]. 

In a medicolegal setting, comprehensive assessment of the cause of injury associated with a traffic crash requires expertise from multiple disciplines. Traffic crash reconstruction and engineering techniques are used to assess the severity and direction of vehicle forces, and typically described in terms of speed change (also known as “delta V”) and principal direction of force (PDOF) applied to the vehicle during the crash. Medical expertise is needed to understand the nature of the collision-related injuries (for both survivable and fatal crashes) and their sequelae in non-fatal crashes. Biomechanical methods provide the nexus between medically documented injuries and the reconstructed crash forces, and epidemiologic methods are used to appropriately categorize and describe the crash and its outcomes, relative to similar collisions.

In civil litigation associated with serious injury and deaths resulting from traffic crashes, a potential topic of dispute is the effect that the presence or absence of a safety device, such as a correctly used seat belt, might have had on the cause of an observed injury, as this may indicate a degree of liability that the claiming party shares for their injuries. While seat belt use provides a benefit in many crash circumstances, this is not always the case. Although it is generally accurate to assert that seat belt use decreases the chance of serious injury and death in traffic crashes [4], it would be obviously incorrect to assume that this means that the use of a seat belt would prevent injury or death in all circumstances. It is well-established that other crash-related factors may be far more important in a specific crash, including the impact severity, crash orientation and type (i.e., rollover vs. non-rollover), direction of impact vector, and the presence and deployment of an airbag [5,6]. As an example, if an unrestrained occupant is thrown clear of a vehicle in a rollover crash and sustains a serious head injury, but the vehicle then burst into flames and completely incinerated, it would be absurd to suggest that the occupant would have fared better if they had been restrained and stayed in the vehicle.

Thus, while the maxim that “seat belts save lives” is generally true, such a generalization sheds no light on important case-specific questions such as “would the unrestrained decedent have survived the 50 mph (80 km/h) crash with a tree, on a more likely than not basis, if he had been using his seat belt?” A reliable answer to such questions requires the analysis of relevant epidemiologic data, in which the risk of a specific injury is quantified and compared for actual (unrestrained) vs. hypothetical alternative (restrained) scenarios.

There are, at present, no published standards for evaluating, quantifying, or characterizing (relative to applicable legal standards) the contribution of a claimant’s failure to use a seat belt to the cause of their injuries. The current discussion is thus designed to set forth a systematic and reliable approach to such an analysis. In the following discourse, methods for providing an evidence-based analysis of the injury risk attributable to seat belt non-use are described and illustrated with a case study of a fatality occurring in an unrestrained driver involved in a high-speed frontal crash. 

**Figure 1:**
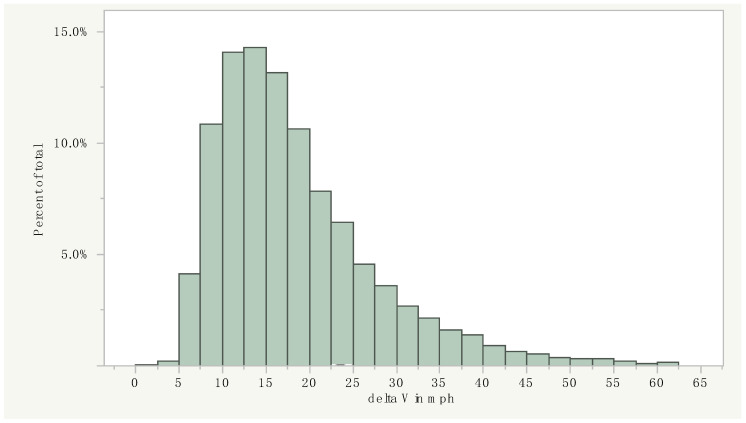
Delta V distribution (in mph) of an estimated 3,552,193 drivers exposed to a frontal crash in US national crash data (the NASS-CDS) for 1990–2014. Source: author.

The case-specific factual elements that are required to perform any similar analysis are as follows:

- *Description of the type of crash*. Seat belts are highly effective at reducing (but not completely eliminating) the risk of ejection from a vehicle, typically during a rollover crash, but provide no substantive reduction in injury risk in a nearside impact (i.e., the injuries to a driver occurring in a driver’s side T-bone crash), as they do not restrict movement toward the B-pillar, side window, and roof rail, and other hard interior vehicle components in such a crash. Similarly, frontal airbags are highly effective at reducing head and face injury risk in frontal crashes, but are designed to not deploy in rear or side impacts, and can even increase injury risk in lower speed crashes if they unintentionally deploy.
- *Quantification of the severity of the crash.* This parameter is quantified by the crash “delta V” or speed change, which is the change in speed of the vehicle occurring over the first approximately 100 msecs of the crash. As a general rule of thumb, seat belt use makes little difference in injury risk in lower speed crashes, the largest amount of difference in moderate to higher speed crashes, and little difference in the highest speed crashes. For example, in a 2 mph (3.2 km/h) delta V frontal crash, there would be no difference between the serious injury risk for a belted vs. an unbelted occupant, as serious injuries do not result from such crashes, regardless of belt use. By the same token, in a 100 mph (160 km/h) delta V frontal crash, the risk of serious injury would be the same for belted and unbelted drivers, as it would be close to 100% for both scenarios. Another factor, when performing a population-based analysis of injury risk by safety device, is the statistical scatter present among the highest and lowest delta V crashes, as the bell-shaped distribution of crashes across the delta V spectrum makes the upper end of the range the least reliable for analysis (see Figure 1).
- *The seating position of the occupant*. In comparison with front seat occupants, rear seat occupants do not have the advantage of an airbag in a frontal collision, and are often closer to rigid interior components of the vehicle, relative to frontal occupants. Additionally, occupants who are out of the usual seating position (i.e., laying down) at the time of a crash may be at particular risk.
- *Detailed description of the nature and severity of the injury*. Some injury types have a high correlation with crash severity and seat belt non-use, where serious head, chest, and abdominal injuries in frontal crashes are prime examples. Other injuries have a poor correlation with either crash severity or seat belt use; an example is spinal disk injuries, which are largely dependent on the pre-crash condition of an occupant’s spine, rather than the severity or orientation of a crash or seat belt use. Other injuries are rare regardless of the severity of the crash or belt use, for example vertebral artery dissection, and there is not enough information to inform a determination of the theoretical effect of a safety device.

The Discussion Section of this manuscript describes how the results of the analysis may be presented in a medicolegal setting, to most accurately quantify the contribution the claimant’s seat belt non-use had on the risk of their injuries, relative to the total risk of injury in the crash. 

## 2. Methods

Analysis of an example from the author’s personal inventory of cases is used to illustrate the application of a systematic approach to estimating the effect of seat belt non-use on the specific injury risk of a litigant by examining the attributable or relative risk of similar injuries in populations of restrained and unrestrained occupants. The approach employs the following steps, which follow the general framework of the INFERENCE causal approach to causation, in which the tenets of epidemiologic causation are combined with knowledge and processes used in forensic medicine and other disciplines to form a systematic process that maximizes reliability, repeatability, and transparency [7]:

1. The legal question at issue requiring analysis is first identified. For the present discussion, the legal question is restricted to the degree that failure to use a seat belt may have contributed to the probability that a claimant sustained a particular injury, and as a corollary, to what extent the claimant contributed to their injury by their pre-crash actions.
2. The nature and severity of the crash is first analyzed using basic principles of crash investigation and crash reconstruction. These include the analysis of evidence gathered from the investigation of the scene and involved vehicles, and the application of primarily Newtonian physics (i.e., momentum, energy, and restitution) via crash reconstruction formulae and software, unique to the facts of each case [8]. When available, the information contained in an airbag control module (ACM) download may provide a higher degree of precision for reconstruction purposes [9]. For planar (non-rollover) crashes, the severity of the crash is quantified using speed change or delta V (in mph or km/h), and the direction of the collision force is based on the reconstructed principal direction of force (PDOF) of the impact, which can be described by using a clock face (with 12 o’clock from the front) or degrees of a circle [8].
3. The nature and severity of the most significant medically observed injuries are described, scaled for severity, and ranked by their Abbreviated Injury Scale (AIS) score in a crash-specific 7-digit identifier called a NASS injury code [10]. The AIS ranks injuries based on their mortality risk, as follows: 1—minor, 2—moderate, 3—serious, 4—severe, 5—critical, 6—maximum, and 7—unknown severity. The typical cut point at which mortality risk is considered more than nominal for an injury is for a serious (AIS 3) injury. It may be of benefit in this step to describe the biomechanical features of the injury of interest, including the mechanism by which it likely occurred during the crash.
4. Data for case-specific injury risk estimation and comparison are accessed from the US crash injury database, the National Automotive Sampling System-Crashworthiness Data System (NASS-CDS) of the National Highway Traffic Safety Administration. The NASS-CDS investigates approximately 5–6000 crashes every year in 24–36 geographic Primary Sampling Units (PSU) at a cost of approximately $US10,000 per investigation. A record of over 800 variables, including weather and road conditions, crash circumstances and severity, restraint use and airbag deployment status, and medically observed injury to occupants, is gathered for each crash by trained NASS crash investigators. In order for a collision to be recorded in the NASS-CDS, it must meet several criteria: a police report was generated, it was located within a primary sampling unit, it involved at least one passenger car, van, or light truck, and at least one vehicle was towed from the crash scene. In turn, these data are weighted to provide a national estimate of all police-reported crashes occurring in the US and involving passenger cars, light trucks, and minivans that were towed due to damage [11].
5. Although the analysis of NASS-CDS data can be accomplished using a variety of software, in the present discussion, statistical analysis of the data abstracted from the NASS-CDS is performed using the SURVEY package included in SAS 9.4 software, in order to account for the complicated sampling structure of the NASS-CDS (SAS Institute Inc., Cary, NC, USA), as this is generally the best practice with these data. The crash and injury case-specific risk that is attributable to restraint non-use is then quantified based on the analysis.

The non-use of a seat belt can only be deemed to be the medicolegal “cause” of a specified injury if it can be shown that more than 50% of the risk of an injury type and severity observed in an unbelted population is eliminated in a belted population. For example, if it was found that unbelted occupants sustain a particular injury 30% of the time in a frontal crash of 25 mph (40 km/h), and belted occupants sustain the same type and severity of injury only 10% of the time in the same type and severity of crash, it could be concluded that the injury risk ratio (also called relative risk, or simply RR) of non-belt to belt use is 3.0 (i.e., the ratio of 30% to 10% is 3.0). The excess, or attributable risk (AR) resulting from seat belt non-use, is quantified via the following formula:

(1) $$\mathrm{AR}=\frac{\mathrm{RR}-1}{\mathrm{RR}}\times 100\%$$

Applied to the 3.0 RR from the above example yields:

(2) $$\mathrm{AR}=\frac{3.0-1}{3.0}\times 100\%=67\%$$

Thus, 67% of the injury risk (i.e., 2.0 out of 3.0 of the risk ratio) is attributable to seat belt non-use. This value exceeds the “more probable than not threshold (>50%)” legal standard to allow for the conclusion that had an unbelted individual with the particular injury been using a seat belt, the injury would have been avoided [12]. 

If the risk ratio does not exceed 2.0, then it cannot be concluded to a >50% probability that the use of the seat belt would have prevented the injury. For example, if the risk of injury in unbelted and belted occupants was 15% and 10% respectively, then the risk ratio between the 2 values would be 1.5. Applying the formula above to the 1.5 risk ratio yields an attributable risk of 33%, substantially less than the 50% more probable than not threshold. This is not to say that the use of a seat belt does not reduce the risk of injury in a similar crash scenario, but rather, when evaluating an individual set of circumstances after the fact, the evidence does not support an opinion that the injury would have been prevented if a seat belt had been used, more than 50% of the time. Statistical tests of significance must accompany the comparison of risks to determine whether the effect of random scatter can be ruled out such that the quantification of the relationships can be considered reliable.

If the risk ratio for non-use of a seat belt exceeds 2.0, then it is the *absolute* risk difference, rather than the attributable risk, that is used to assess the contribution of the claimant’s failure to use a seat belt to the cause of their injuries. Using the values in the first example above, in which the attributable risk associated with the 3.0 risk ratio was calculated as an AR of 67%, the absolute risk difference would be 20%, based on the fact that the rate at which unbelted occupants are injured (30%) is only 20% more than the rate at which belted occupants are injured (10%). The principles and rationale underlying this approach are more thoroughly described in the Discussion Section of the paper.

## 3. Results

The following case study illustrates the application of the methods described in the previous section. Personal identifiers have been redacted or changed from the case file, and unique aspects of the case with high relevance to the analysis have been emphasized. Although the author was contacted by counsel for the decedent’s family, the design and results of the analysis were not dependent on the legal theory of the retaining counsel. During the legal proceedings, all data and statistical code used for the analyses were made available to the opposing side, to foster transparency of methods.

### 3.1. Case Description 

**Figure 2:**
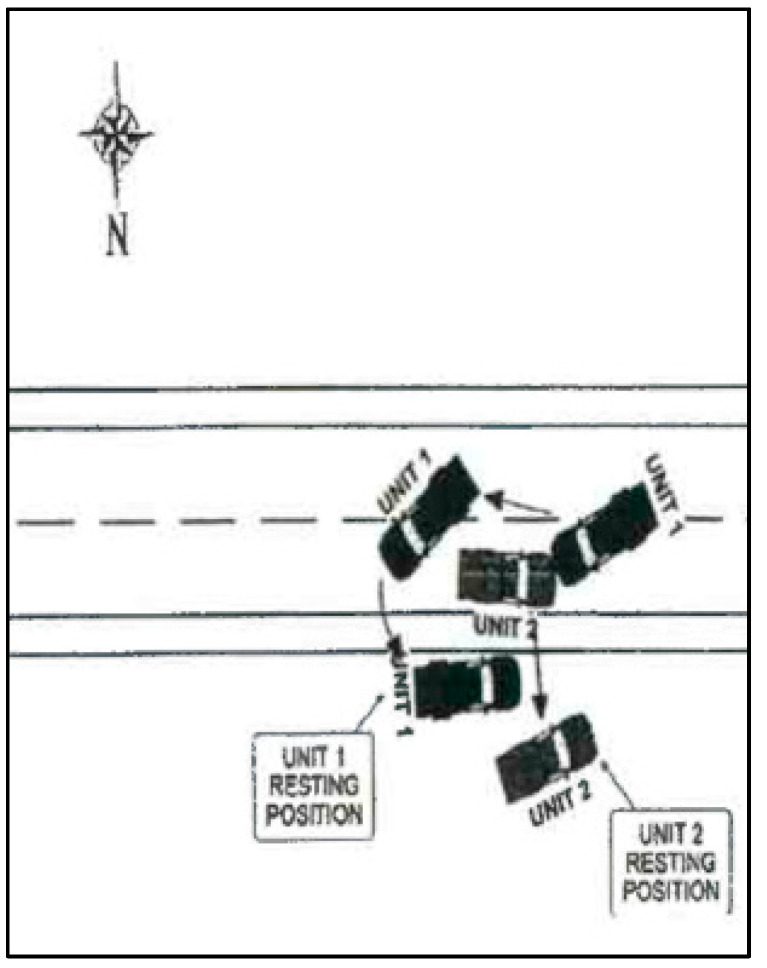
Police diagram of the crash. The decedent’s Chevrolet is “Unit 1”.

**Figure 3:**
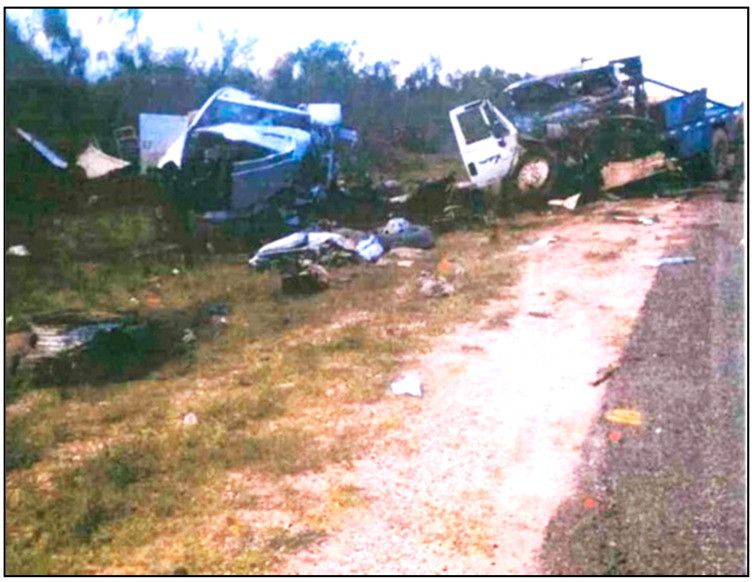
Scene photo depicting final rest for both vehicles.

**Figure 4:**
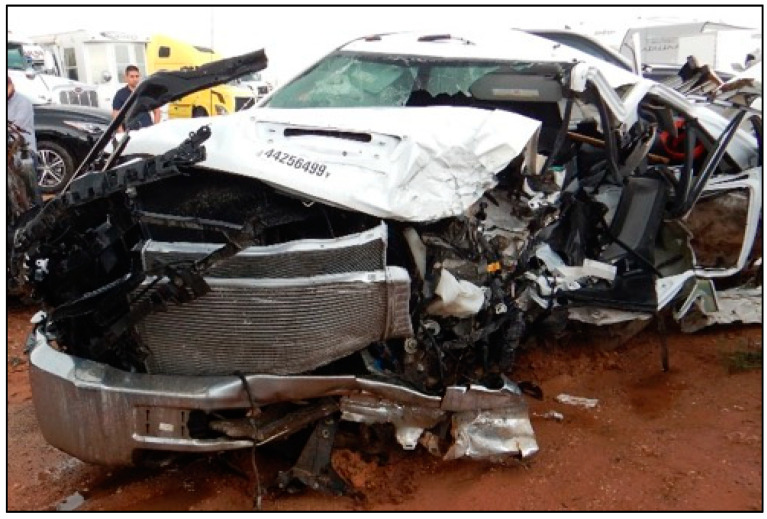
The decedent’s Chevrolet post-collision (photograph taken at a wrecking yard).

**Figure 5:**
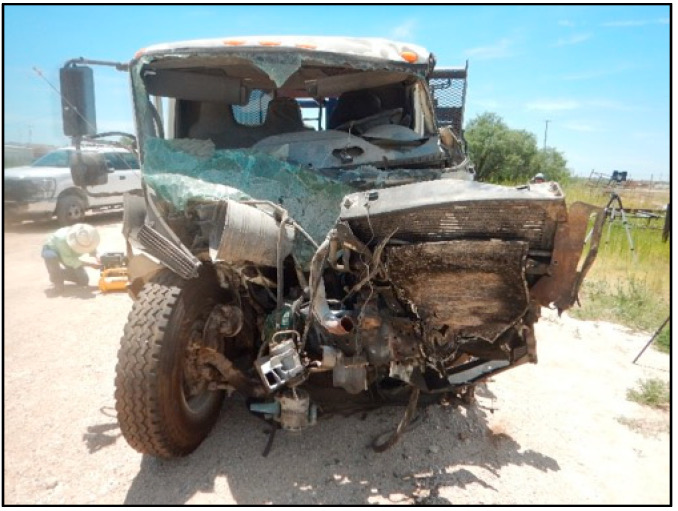
International truck, post-collision, front view.

**Figure 6:**
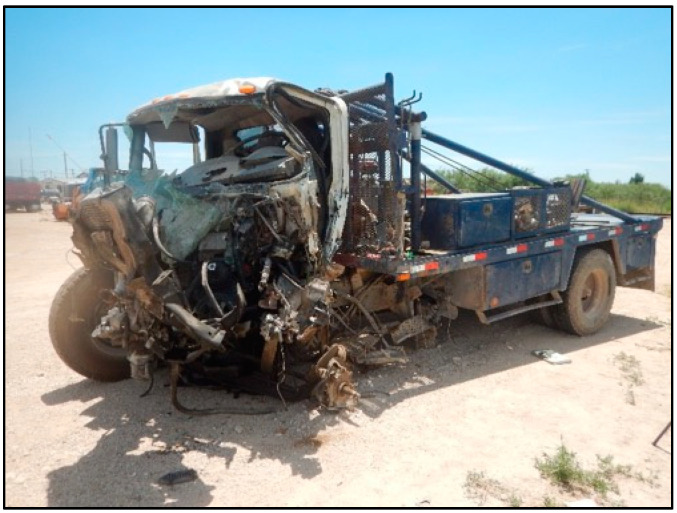
International truck, post-collision, left front ¾ view.

The decedent, a 22-year-old male, was the unrestrained driver of a 4-door Chevrolet Silverado 3500 pickup traveling West at highway speed on a rural 2-lane road (designated “Unit 1” in Figure 2). The vehicle (Unit 1) was struck on the left front/side by the left front of an International heavy truck (Unit 2 in Figure 2), that had been traveling the opposite direction and crossed the center line and entered the decedent’s lane of travel. The decedent was killed outright by the crash, and could not be resuscitated after he was extricated from the vehicle. Figure 2, Figure 3, Figure 4, Figure 5 and Figure 6 depict a police diagram and photos of the subject vehicles post-collision.

The photographs demonstrate that the Chevrolet Silverado sustained more than approximately 4 feet of crush damage, extending from the left front to the left rear side. The PDOF (principal direction of force) of the crash was from the front left, or approximately the 10:30 to 11 o’clock direction. The driver’s side front airbag had deployed, and the occupant space on the driver’s side was severely compromised. 

The crush damage to the International truck largely mirrored the damage to the Chevrolet, and included more than 5 feet of crush to the left front extending beyond the left front wheel well, with induced (non-contact) damage from the front and into the cabin. The PDOF to this vehicle appeared to be close to that of the Chevrolet, from approximately the 11 o’clock direction.

The autopsy of the decedent revealed chest and abdominal injuries, including an un-survivable complete aortic transection with associated bilateral hemothoraces [13], bilateral rib fractures, and pulmonary contusions, as well as lacerations of the liver and spleen with associated hemoperitoneum. Although there were no skull fractures, subarachnoid hemorrhage and cerebral edema were observed, along with lacerations and abrasions of the head and neck. There was also a comminuted fracture of the left femur and left shoulder dislocation noted. A toxicological analysis of the decedent’s blood revealed the presence of benzoylecgonine (a cocaine metabolite) at 99 ng/mL, which indicated that the decedent had been exposed to cocaine at some time during the 5 days preceding the crash, but not that the decedent was impaired by cocaine at the time of the crash. The contemporaneous law enforcement investigation of the crash did not find any evidence of impaired driving by the decedent.

### 3.2. Reconstruction of the Crash

The reviewed evidence indicated that the eastbound International truck crossed the oncoming lane at highway speeds and struck the westbound Chevrolet Silverado on the westbound shoulder of the highway. The collision was essentially head-on, with a sustained left side impact to the Chevrolet Silverado. 

An inspection of the vehicles indicated that the reason the International truck suddenly crossed into the westbound lanes and caused the crash was due to the failure of the left front tire of the vehicle, which was found to be excessively worn. An airbag control module (ACM) download was performed on the decedent’s Chevrolet Silverado, resulting in a retrieved airbag deployment event. The report indicated the Chevrolet Silverado was traveling 58 mph (93 km/h) at ½ s prior to the crash, with the brakes activated. The data indicated that the Chevrolet Silverado was subjected to a total delta V of approximately 55.8 mph (89 km/h) with a peak total acceleration of 71 g, putting the crash in the upper 1% of frontal crashes in the US for severity (see Figure 1, above) [14].

### 3.3. Seat Belt Non-Use Attributable Risk Analysis

As the decedent was killed in the subject collision, the outcome of interest for the analysis was death. Data were accessed from the NASS-CDS database, specific to the following parameters: included were all drivers that were exposed to a single frontal collision, with a direction of force ranging from 9 to 11 o’clock (to account for the fact that the vehicle damage was primarily focused on the driver’s side of the vehicle and at the decedents’ position). Only cases in which seat belt use or non-use was known, and where it was known if there was a driver’s side frontal airbag deployment, were included. Crashes that resulted in a rollover were excluded. The outcome of interest was death, including deaths that occurred up to 30 days after the crash. The analysis utilized the years 2001–2015 (15 years, inclusive).

A logistic regression model was constructed from the results of the analysis. This statistical method allows for the modeling of a binary outcome (e.g., death vs. survival, injury vs. no injury) over a continuous variable (e.g., delta V, number of vehicle rolls), while accounting for the effects of covariables (e.g., vehicle type or year, airbag deployment). The model used for the case analysis accommodated the effect of covariables on the outcome of interest (death), aside from the two main variables of interest, which were delta V and seat belt use. Other potentially predictive factors that were accounted for, specific to the circumstances of the decedent’s crash, were the fact that he was driving a pickup truck, the airbag in the vehicle deployed, the decedent was not ejected, and the year of manufacture of the Chevrolet pickup.

The results of the analysis were as follows: there were a weighted total of 2,272,756 belted and 669,097 unbelted drivers used for the logistic regression model. Among the belted drivers, there were 592,311 with an airbag deployment, and 99,579 unbelted drivers with an airbag deployment.

**Figure 7:**
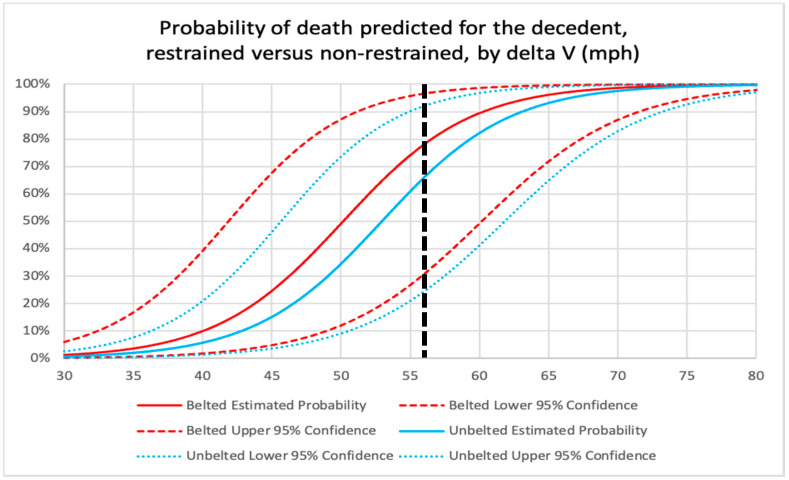
Chart depicting results of the logistic regression analysis, with risk curve for death by delta V, for restrained vs. unrestrained drivers.

The results of the logistic model are presented in the chart in Figure 7, with the dashed straight line demonstrating the risk of death for a belted (red solid line) and unbelted (blue solid line) driver at delta V’s ranging from 30 to 80 mph, with 56 mph (the delta V for the subject crash) indicated with a vertical black dashed line. The 95% confidence intervals (the dashed red and blue lines) demonstrate the amount of statistical scatter among the data.

At 56 mph (89 km/h) delta V, it can be seen that the solid red line representing the death risk for belted drivers intersects with the dashed black line at 78% and the solid blue curved line, representing the death risk for unbelted drivers, intersects at 66%. These results indicate that belted drivers are at higher risk of death, in comparison with unbelted occupants, in very high-speed frontal crashes like the subject collision. This finding may be entirely due to the relatively small number of high-speed crashes like the subject collision in the NASS-CDS data and the resulting large amount of statistical scatter in the data, as indicated by large degree of overlap of the 95% confidence intervals in the chart in Figure 7. Another potential explanation for the seemingly counterintuitive finding of higher death risk for belted occupants in higher speed crashes is that much of the differential benefit of seat belt use is lessened by the protection afforded by an airbag. Additionally, unbelted drivers are more likely to be ejected into the passenger side of the vehicle and away from the driver’s side, where the greatest occupant compartment intrusion is occurring. 

Regardless of the explanation, the difference in risk of death is not statistically significant, which is indicated by the overlap of the confidence intervals. Thus, after reviewing all of the results of the analysis, it must be concluded that there is no statistically significant difference in the risk of death for a driver who is belted vs. unbelted. 

The finding that a seat belt would have made no difference on the survivability of the subject crash applies directly to the decedent. From a biomechanical perspective, the level of forces applied to his body by the crash (71 g was applied to the vehicle, based on the ACM download) were exceedingly high, and the high degree of crush to the driver’s side occupant space would have magnified the collision forces as they were delivered to the decedent’s body. These forces and intravehicular events would have overwhelmed any potential protective capacity of a shoulder and lap belt in the subject crash. The evidence supporting this conclusion is the fact that, regardless of seat belt use and airbag deployment, there is a more than 50% risk of death in a 56 mph (89 km/h) delta V frontal crash. This means that the decedent would have died in the subject crash, on a more likely than not basis, *regardless of seat belt use.*

Based on the analysis, it was concluded, for purposes of the legal action, that the decedent’s seat belt non-use did not contribute to his risk of death in the subject high-speed left frontal crash.

## 4. Discussion

Traffic crashes may result in legal claims when injuries occur in occupants who did not cause the crash [15]. Epidemiologic (population-based) issues are commonly encountered in crash-related litigation, most often relating to causation of injury, and in particular the “but-for” scenario as it relates to the use or presence of a safety device, or possible design defect. Examples include the potential effect that an unused seat belt may have had on the specific injury risk to a plaintiff making a claim for injury in a crash caused by another motorist, or the injury risk difference attributable to an airbag that failed to deploy when it should have, or when a roof has crushed excessively during a rollover crash. Another term for the “but-for” scenario is *counterfactual causation,* the process of comparing the risk of injury from a potentially harmful event vs. the risk of the same injury at the same point in time, but in the absence of the potentially harmful event, given what is known about the individual’s condition and circumstances of the exposure [16,17].

This type of approach, which relies to a varying degree on statistical analysis of epidemiologic data, is used in relatively unique circumstances and typically only in a medicolegal or research setting. It is distinct from the typical intuitive approach to causal analysis in traffic crash-related injury documentation and investigation, which is clinical reasoning based on observation, and a common sense approach that accounts for the history of the injury [7]. Outside of a forensic medical analysis, causal assessment of crash-related injuries and death are largely based on a history-based assessment of temporal order and proximity between a crash and an injury, i.e., if an observed and plausibly associated injury follows a crash relatively closely in time, then the injury is attributed to the crash. 

Clinical causation of serious injuries following a traffic crash is also largely based on the high degree of correlation between the injury and a high-energy, extra-physiologic event. As an example, for a femur fracture observed in a front seat occupant following a moderate speed frontal crash, the injury is highly correlated with the type of sudden axial bending load to the thigh that can result from a frontal collision, even one occurring at relatively low speed [18]. An additional consideration that accompanies clinical reasoning is the specificity of the injury to the injury mechanism of a traffic crash. A high degree of specificity is why, despite the dissimilarity in severity, both aortic transections and whiplash injuries are closely associated with traffic crashes. Even without knowing the history of either injury, the diagnoses serve as a strong indication that a traffic crash was the cause. 

In civil litigation involving traffic crash-related injury and death, it is reasonable to raise the non-use of a seat belt as a theory of contributory negligence by a claimant. As demonstrated by the case analysis presented in the current discussion, the evidence-based investigation of the validity of such an allegation of contributory negligence can be technically complex, and requires a range of skills and knowledge from a variety of disciplines, including crash reconstruction, injury biomechanics, medicine, epidemiology, and even biostatistics. 

Aside from the case-specific analysis methods described above, another factor to consider in such disputes is the nature and wording of the legal question at issue. If there is a legal requirement that the use of a safety device would, on a “more likely than not” basis have prevented an injury, then there is a quantifiable threshold that must be met for an opinion to be considered relevant. The more likely than not civil litigation standard refers to the need to meet a >50% threshold to demonstrate a causal relationship. 

The metric of risk that relates to causation is the amount of risk that is attributable to the adverse exposure of interest in a relative risk ratio. As an example, if epidemiologic data indicate that a serious head injury occurs in a two-full-turn rollover crash in 13% of belted drivers, and 27% of unbelted drivers, the excess injury risk among the unbelted drivers that results from non-use of a seat belt is (27% − 13% = 14%). As 14 is 52% of 27, the percent of the total risk of serious injury that can be attributed to non-use of a seat belt is 52%. Thus, in a medicolegal setting, it is accurate to state that among unbelted drivers who sustain a serious injury in a two-turn rollover crash, the injury would not occur, on a more likely than not (>50%) basis, if a belt had been used. The same risks can be compared as a risk ratio. Using the values from the above example, the ratio of non-use to use of a seat belt is ((27/13) = 2.1). 

Although the values used for the example above would seem to indicate that the failure to use a seat belt caused the injury, the actual meaning of these values, as they might be used to quantify the contributory negligence of the injured party in a legal action, is more nuanced. This is largely related to the proximity of the failure to use a seat belt to the cause of the injury. This concept can be explained by an example. If a seat belt latch fails due to a manufacturing defect during a rollover crash, and a driver is ejected and killed during a rollover crash, an analysis that indicates a 10% ejection and death risk for an unbelted occupant vs. a 1% risk for a belted occupant would indicate a 90% reduction in the risk of death if the seat belt latch had not failed (i.e., 9% out of 10% of the total risk of death is attributable to the failure). There are no intervening events between the failure of the seat belt and the event leading to the death, which is the ejection and associated injuries, and thus the cause and effect are proximate. If the risk is translated to 100 occupants (vs. percentage), then the only groups we are interested in are the 10 deaths among the unbelted occupants vs. the 1 death among the belted occupants. The surviving occupants are not considered in the analysis because the population the occupant belongs to is those occupants who are ejected and who die.

This is not the case when attempting to quantify the causal contribution of a claimant’s decision to not use a seat belt to the risk of an injury or death. There is an additional risk that intervenes between the seat belt non-use and the injury, and that is the total risk from the crash. This is because the “exposed” population the decedent belongs to is those occupants who do not use a seat belt, and who are involved in a crash. Thus, unlike the example above, we must consider all outcomes from the crash among the unbelted (90 survivors and 10 deaths), which is compared to all outcomes from the crash among the belted (99 survivors and 1 death). It is, therefore, the absolute difference in risk that quantifies the degree of contributory negligence of the claimant by failing to use a seat belt, which, from the example above, is 9%, rather than 90%. The logic of using absolute risk difference to apportion risk between the claimant for failing to use a seat belt, and the defendant for causing the crash, can be illustrated with an extreme example, in which an injury occurs in only 10 out of 10 million unbelted drivers, and in 1 out of 10 million belted drivers. It is easy to see that it would be improper to use the attributable risk of 9 out of 10 injuries eliminated by seat belt use to characterize the contributory negligence of the claimant, when the decision not to use a seat belt only increased the risk by 9 out of 10 million, or less than 1 in a million crashes.

Based on the preceding discussion, a forensic analysis of contributory negligence secondary to seat belt non-use should proceed by first assessing whether there is statistically reliable epidemiologic evidence that the **attributable risk** of non-use is greater than 50% (if the legal question requires that a “more probable than not” (>50% probable) standard be met). If this first threshold is met, then the amount to which the claimant’s seat belt non-use contributed to the overall risk of injury should be quantified in terms of **absolute risk**.

## 5. Conclusions

The evidence-based evaluation of the degree to which seat belt non-use contributed to the risk of injury or death of a claimant is an involved and, to some degree, complex process. The analysis requires the combination of crash reconstruction, biomechanical, medical, and epidemiologic/biostatistical principles and techniques, in order to arrive at an accurate (i.e., precise and reliable) determination of contributory negligence, suitable for presentation in a medicolegal setting.

## Data Availability

The police report that served as the source material used for Figure 2, Figure 3, Figure 4, Figure 5 and Figure 6 can be found at https://cris.dot.state.tx.us/public/Purchase/app/search/form/detail. The national crash injury data analyzed for this study are available from https://www.nhtsa.gov/file-downloads?p=nhtsa/downloads/NASS/.

## Abbreviations

NASS-CDS	National Automotive Sampling System-Crashworthiness Data System
PDOF	Principal Direction of Force
AIS	Abbreviated Injury Scale
